# A governmental program to encourage medical students to deliver primary prevention: experiment and evaluation in a French faculty of medicine

**DOI:** 10.1186/s12909-020-02472-z

**Published:** 2021-01-13

**Authors:** Enora Le Roux, Marta Mari Muro, Kore Mognon, Mélèa Saïd, Viviane Caillavet, Sophie Matheron, Séverine Ledoux, Philippe Decq, Florence Vorspan, Yann Le Strat, Constance Delaugerre, Morgane Le Bras, Corinne Alberti, Philippe Ruszniewski, Philippe Zerr, Albert Faye

**Affiliations:** 1 Université de Paris, ECEVE UMR 1123, Inserm, F-75010 Paris, France; 2 Service de Santé Publique, AP-HP, Nord-Université de Paris, Hôpital Universitaire Robert Debré, Unité d’épidémiologie clinique, Inserm, 48 boulevard Serurier, CIC 1426, F-75019 Paris, France; 3 General Medecine department, Université de Paris, F-75018 Paris, France; 4 GHT NOVO, Nursing school administration, F-95300 Pontoise, France; 5 Unité UMR 1137 IAME, Université de Paris, Inserm, F-75010 Paris, France; 6 AP-HP, Nord-Université de Paris, Hôpital Universitaire Bichat-Claude Bernard, Service de maladies infectieuses et tropicale, F-75018 Paris, France; 7 AP-HP, Nord-Université de Paris, Hôpital Louis Mourier, Service des explorations fonctionnelles, F-92700 Colombes, France; 8 AP-HP, Nord-Université de Paris, Hôpital Beaujon, Service de neurochirurgie, F-92110 Clichy, France; 9 AP-HP, Nord-Université de Paris, Hôpital Lariboisière, Service de psychiatrie, F-75010 Paris, France; 10 AP-HP, Nord-Université de Paris, Hôpital Louis Mourier, Service de psychiatrie, F-92700 Colombes, France; 11 INSERM UMR1266, Institute of Psychiatry and Neurosciences of Paris (IPNP), F-75014 Paris, France; 12 AP-HP, Nord-Université de Paris, Hôpital Saint Louis, Service de virologie, INSERM U944, F-75010 Paris, France; 13 Université de Paris, UMR_S 976, Inserm, Institut de Recherche Saint Louis, F-75010 Paris, France; 14 Dean of Faculty of medicine, Paris Diderot University, F-75010 Paris, France; 15 Service de Pédiatrie Générale, AP-HP, Nord-Université de Paris, Hôpital Universitaire Robert Debré, F-75019 Paris, France

**Keywords:** Medical students, Prevention, Education, Implementation, Process evaluation

## Abstract

**Background:**

A public health student service was set up by the French government in 2018 with the aim of increasing awareness of primary health promotion among the 47,000 students of medicine and other health professions. It is an annual program involving community-based actions on nutrition, physical activity, addiction or sexuality. Our objective was to evaluate its implementation at local level and the different experiences of the stakeholders.

**Methods:**

A quasi-experimental study using process evaluation was performed in a Faculty of Medicine in Paris. Quantitative and qualitative data were collected from medical students who carried out preventive health actions, in the institutions in which the actions took place and from a subsample of beneficiaries.

**Results:**

One hundred and eight actions were carried out by 341 students in 23 educational or social institutions, mostly high schools (*n* = 12, 52%). Two thirds of the students did not feel sufficiently prepared to deliver preventive health interventions (65.7%, 224/341); however the beneficiaries found that the interventions were good (278/280, 99,2%). Nineteen (83%) of the host institutions agreed to welcome health service students again, of which 9 required some modifications. For students, the reporting of a satisfactory health service experience was associated with the reporting of skills or knowledge acquisition (*p* < 0.01). Delivering actions in high schools and to a medium-sized number of beneficiaries per week was associated with students’ satisfaction. No effect of gender or theme of prevention was observed. For 248/341 (72.7%) students, the public health service program prompts them to address prevention issues in the future.

**Conclusion:**

The public health service undertaken by medical students through the program is a feasible and acceptable means of delivering preventive actions. Reinforcement of training and closer interaction with the host institutions would improve results.

**Supplementary Information:**

The online version contains supplementary material available at 10.1186/s12909-020-02472-z.

## Background

Non-communicable diseases (e.g. cardiovascular diseases, cancers, respiratory diseases and diabetes) account for the vast majority of the global burden of disease in the Organisation for Economic Co-operation and Development (OECD) countries, and are expected to increase further in the context of aging societies [1]. These diseases can be prevented by effective interventions targeting exposure to major risk factors such as smoking, unhealthy diets, sedentary lifestyles and substance abuse [2].

However, current resources are inadequate to the needs. OECD countries allocate on average less than 3% of their total health expenditure to public health and preventive health activities [3]. In addition, in many countries, medical studies do not fit with the goal of making clinicians health promoters [4].

A number of initiatives have been carried out in different parts of the world to address and improve this situation. Recently, American medical educators have been tasked with putting more emphasis on prevention, population care, public health and community medicine [5]. Two experiments in prevention interventions delivered by American and German volunteer students in schools [6, 7] have shown their cost-effectiveness. Against this background, the national Public Health Service program (*service sanitaire* in French) was set up by the French government in 2018/2019. Its plan involves about 47,000 health students per year carrying out practical exercises in health promotion or primary prevention during their initial training, mainly aimed at young people on priority prevention themes: nutrition, physical activity, addictions, and sexual health. The frequency of risky behaviours remains high in France, in particular among young people and disadvantaged social groups [8].

The initiative was developed to accompany the reduction of risky behaviours, to reduce social inequalities in health by carrying out preventive actions throughout France, and to modify the practices of new health professionals - all for a reasonable financial investment.

Our objectives were (i) to evaluate the implementation of the public health service by a faculty of medicine in its first year of deployment, (ii) to assess the experiences of students taking part in the program of service, of the beneficiaries of the actions, and of the host institutions, and (iii) to identify the factors related to student satisfaction.

## Methods

A quasi-experimental study with process evaluation was performed during the academic year (September 2018 – June 2019) in “Denis Diderot” Faculty of Medicine (Université de Paris), Paris, France.

### Health service intervention

**Fig. 1:**
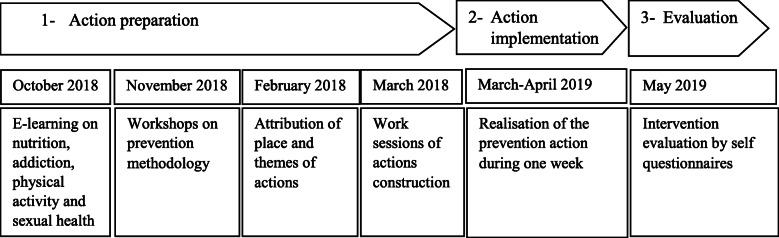
Key steps of the health service intervention towards medical students

A steering committee was created to implement the public health service locally; it involved professors teaching in the faculty of medicine or school of nursing, student representatives, and the evaluation team. The committee was responsible for the global organisation and smooth functioning of the service, the training and tutoring of students in topics proposed by the government and the faculty, and the recruitment of the host institutions. The decision to carry out the public health service program in the 3rd year of medical studies was taken by the government, probably because of the training schedule (in their 2nd and 3rd year, students learn the theoretical clinical basis required for preventive health action, and in the 4th year, students begin their attachment to a a hospital). All non-repeating third-year medical students had to perform their public health service which was based on 3 steps: preventive action preparation helped by teachers, action implementation (in groups of 3 to 4) and evaluation (Fig. 1). In each host institution, a reference person (e.g a life science teacher) was identified to be the students’ contact. He/she had to guide the development of the health promotion or preventive action according to the population characteristics, and to ensure its proper implementation in the institution (communication to the beneficiaries of the action, provision of a suitable space and equipment for students...). The Template for Intervention Description and Replication (TIDieR) [9] was used to describe the program (a studied intervention) and its local implementation (Supplementary material 1).

### Process evaluation

In order to assess the feasibility and acceptability of the public health intervention and the satisfaction associated with it, mixed methods were used according to the recommendation for process evaluation of complex interventions [10]. Quantitative and qualitative data were collected via observations of the preparation phase, and via semi-structured interviews and questionnaires developed for this study (Supplementary material 2).

Seven groups of students were recruited on a voluntary basis to participate in the qualitative study based on observation of the preparations and implementation of their own actions. These groups had to deliver actions in two host institutions (a high school providing general and vocational education, and a university with multidisciplinary training in the departments of humanities and social sciences), which were chosen after agreement by their directors and selected for their diversity in beneficiary profiles and plurality of prevention themes addressed by the actions. A seventh year medical student specialising in public health carried out the observation and assessed the quality of the actions based on grids grouping regional institutions of education and health promotion in France. Other questionnaires used in the evaluation were developed as part of the study. An anonymous 14-item satisfaction questionnaire in paper format was made available to the beneficiaries of the observed actions; they were invited to complete it immediately after the action. Completion was voluntary. One month after the intervention, all the medical students filled out an 82-item web-based questionnaire assessing the training, the prevention action preparation, its implementation and their overall experience of the public health program. The reference persons in the host institutions were asked by email, one month after the completion of the last prevention actions, to complete an 11-item questionnaire on their experience.

Data were collected from March 2019 to June 2019. As an education quality improvement project, this study does not require Institutional Review Board approval according to French regulations (Regulation (Eu) 2016/679 Of The European Parliament And Of The Council of 27 April 2016 and repealing Directive 95/46 / EC (General Data Protection Regulation)).

### Data analysis

Qualitative data were transcribed, synthesised and analysed by themes. Names associated with quotes are fictitious. Quantitative data were described as numbers (percentages) for categorical variables and as medians (1st quartile-3rd quartile) for continuous variables. The statistical association between student satisfaction (binary) and other factors was tested using logistic regression. To select variables to include in the multivariate analysis we first performed univariate analysis and included in the model all variables with a *p* value < 0.20 (sensitivity analysis was done with p value< 0.10, with which the final model was also obtained). Statistical associations were considered significant when *p* < 0.05. All quantitative analyses were performed with SAS v9.4 (SAS Institute Inc.; Cary, NC: USA). Triangulation, combining the results of qualitative and quantitative methods, was used at the time of analysis and interpretation [11].

## Results

Of the 358 medical students eligible for the public health service program in our chosen faculty, 341 participated in the evaluation and achieved 108 different prevention actions. A total of 23 institutions agreed to host the health service actions: one middle-school, twelve high schools, seven universities and three social organisations dedicated to migrants and homeless people. The majority of students delivered their action to 50 to 150 beneficiaries per week, whose median age was 18 (16.5–21.5). Intervention themes and contents are detailed in Table 1.

**Table 1:** General characteristics of the 108 interventions

Intervention theme		
		***N students (%)***
***Nutrition***		**79 (23.2)**
Specific subtheme(s) (optional)		26
	*Good quality food*	15 (57.7)
	*Cardiovascular Risk Factors*	10 (38.5)
	*Dental health*	1 (3.8)
***Physical activity***		**68 (19.9)**
Specific subtheme(s) (optional)		18
	*Overweight / obesity*	4 (22.2)
	*Sports*	3 (16.7)
	*Others*	11
***Addiction***		**88 (25.8)**
Specific subtheme(s) (optional)		56
	*Alcohol*	44 (78.6)
	*Tobacco*	33 (58.9)
	*Drugs*	18 (32.1)
	*Screen*	7 (12.5)
	*Food habits*	2 (3.6)
***Sexuality***		**106 (31.1)**
Specific subtheme(s) (optional)		70
	*Sexually transmitted infection*	46 (68.7)
	*Contraception*	40 (59.7)
	*Sexual consent*	9 (13.4)
	*Termination of pregnancy*	7 (10.4)
**Intervention components**		
	*Discussion/Counselling*	80 (74.0%)
	*Oral presentation*	74 (68.5%)
	*Questionnaires/survey*	67 (62.0%)
	*Challenges/serious games*	31 (28.7%)
	*Clinical measures*	13 (12.0%)
	*Rewards or goodies*	12 (11.1%)
	*Delivery of written information*	12 (11.1%)
		***Med (Q1-Q3)***
**Number of components by intervention**		2.5 (1.5; 3.5)
**Intervention duration (minutes)**		45 (10; 60)

Among the 108 groups of students, 7 were invited to participate in observation and 6 accepted (the seventh group who did not agree to be observed did not deliver any prevention action, apparently due to lack of motivation and preparation). For the prevention action preparation, students took advantage of the faculty’s work sessions to meet members of their groups and plan a large part of the action; during this time they could benefit from teacher support and e-learning resources.

“The pre-action work session was good, it was really focused on the theme, and it was good to meet with other groups that had the same theme. It helped us a lot, it was concrete, we started working on the project, they [the teachers] corrected us, they offered us ideas, that was good.” - Agnès.

“I was less worried after work session because it helped us figure out what to say. We started with an idea and he [the teacher] advised us to do it differently so there you have it, that helped us.” - Noémie.

Then students organised via Facebook / e-mail with an additional meeting to finalise the content of their action. Action implementation in the Social Sciences university department was held in the entrance hall; the supervisor (a professional employee of the administration) was mainly present to deal with the logistical questions of the students who were therefore in autonomy during the action.

“I think it’s the audience that scares us a little. If we were in their position: if there are students standing there in the hall of our faculty, I don’t know if I am stopping “- Axelle.

In the high school, the actions were held in classrooms and supervised by teachers; the main supervisor planned the agenda and logistics (programming the places, times and groups of pupils benefiting from the actions) and most asked for a meeting with the students before the action.

“Another constraint, the school director who absolutely wants to meet us when it was not the week dedicated to health service yet... We already take the time to prepare everything, meet, and go there ... it adds a half-day of work.” Charly.

The observation showed that all groups delivered valid information, all the contents were relevant in relation to the themes and the target population and the beneficiaries were made active. However, contrary to what is recommended for the implementation of prevention actions, the objectives of the action were not always announced and some students provided shallow / incomplete information (e.g. “cannabis in itself is not dangerous”). With one exception, none induced negative feelings among the beneficiaries. Negative reactions, of discomfort, were observed during some exchanges with female students during an action concerning sexual health led by a group composed of 3 male students. The main facilitator identified was the use of quizzes or question boxes to launch the prevention action and adapt it to the public; the main obstacle was time management.

Satisfaction questionnaires were collected from a random sample of 137 high school pupils and 143 social science student beneficiaries: 265/280 (95.3%) declared that the action was well adapted to their level of knowledge, 268/280 (97.8%) that teaching methods were well adapted, 241/280 (87.3%) that the duration was satisfactory, 269/280 (96.8%) that it was well adapted for asking questions. Overall 267/280 (99,3%) beneficiaries ranked the prevention action as “good” (93/280–34.6%) or “very good” 174/280 (64.7%). An improvement in knowledge was reported by 248/280 (93.6%) of the respondents, and 201/280 (77.3%) stated that it encouraged them to change their own health behaviour, whatever the chosen theme.

Among the medical students, final evaluation revealed that 184/ 341 (54%) had a very satisfactory or satisfactory experience of the public health service. The training delivered during the action preparation was perceived as useful by 137/341 (40.2%) in terms of e-learning; by 213/341 (62.5%) in terms of the workshops about prevention methodology; and by 272/341 (79.8%) in terms of the supervised work session dedicated to the action elaboration. However, the organisation set up by the faculty to support students in their health service was deemed “not sufficient to be well prepared for the prevention action” by 224/341 (65.7%), mainly due to the lack of time allocated to prepare the action. According to students, the reference persons in the host institutions helped 205/341 (60.1%) of them in their action preparation. The median amount of time students dedicated to preparing their action was 5 h (:3–7). For 68/341 (19.9%) the workload associated with the health service program was excessive. When implementing their prevention actions, 263/341 (77.1%) students declared that the allocated places in the structure were appropriate, 275/341 (80.6%) had all the necessary materials at their disposal and 300/341 (88.0%) retrospectively reported that the educational method they chose was adapted.

“There were major organizational and implementation concerns, both in terms of the resources offered and the process as well as in the motivation of the students to carry out this service, but it was an extremely interesting experience on a personal level in relation to exchanges with the beneficiaries - in the content and the way of adapt speech- and the reflection on prevention in general.” Leo.

“For me it would be up to social professions or associations to carry out this prevention, because our knowledge certainly allows us to give information to beneficiaries but we have not received any information or training concerning how to get in touch with beneficiaries and this health service was more about collaring people to interest them, which is not necessarily easy in front of students who do not necessarily want or do not have the time. [...] The format in the schools where the action consists in speaking in classes seems to me more adapted.” Elsa (who delivered her action in University).

“I think this health service is a good idea and can really lead to something positive. However, I see several negatives aspects. I think that as medical students, we don’t have the time to devote a whole week to do prevention, which is moreover unpaid. [...] We inquired about the subject in advance, but we are not yet doctors! I have 1 or 2 times realized that I had given false information to a beneficiary (on contraception, or sexual practice).” Cécile.

Perceptions of positive impact of the program and characteristics of students who reported them are presented in Table 2. Four impacts were particularly studied: willingness to change health behaviour, acquisition of medical knowledge and prevention skills, and ease in addressing prevention issues in the future.

**Table 2:** Characteristics of all students participating in the public health service program and those who reported personal positive impact (groups are not mutually exclusive)

	***All students*** ***(n = 341, 100%)***	Students who are willing to change behavior (*n* = 111, 32.5%)	Students who acquired knowledge on the theme (*n* = 194, 56.9%)	Students who acquired new skills in prevention (*n* = 240, 70.4%)	Students who declare ease in addressing prevention issues in the future (*n* = 248, 72.7%)
	***N (%)***	***N (%)***	***N (%)***	***N (%)***	***N (%)***
**Gender** (Missing data = 5)					
***Male***	105 (30.8)	36 (32.4)	54 (27.8)	72 (30.0)	74 (29.8)
***Female***	236 (69.2)	75 (67.6)	140 (72.1)	168 (70.0)	174 (70.2)
**E-learning participation**	216 (63.3)	64 (57.6)	116 (59.8)	152 (63.3)	158 (63.7)
**Place of intervention**					
***Middle School***	13 (3.8)	2 (1.8)	5 (2.6)	11 (4.6)	12 (4.8)
***High school***	143 (41.9)	60 (54.0)	107 (55.2)	118 (49.2)	122 (49.2)
***Universities***	159 (46.6)	42 (37.8)	71 (36.6)	86 (35.8)	92 (37.1)
***Social structure***	26 (7.6)	7 (6.3)	11 (5.7)	25 (10.4)	22 (8.9)
**Intervention theme**					
***Nutrition***	79 (23.2)	32 (28.8)	47 (24.2)	55 (22.9)	58 (23.4)
***Physical activity***	68 (19.9)	20 (18.0)	29 (14.9)	46 (19.2)	49 (19.8)
***Addiction***	88 (25.8)	28 (25.2)	51 (26.3)	60 (25.0)	59 (23.8)
***Sexuality***	106 (31.1)	31 (27.9)	67 (34.5)	79 (32.9)	82 (33.1)
**Number of beneficiaries per week**					
***< 10***	33 (9.7)	6 (5.4)	7 (3.6)	10 (4.2)	14 (5.6)
***10–50***	108 (31.7)	27 (24.3)	48 (24.7)	62 (25.8)	66 (26.6)
***50–150***	137 (40.2)	50 (45.0)	98 (50.5)	117 (48.7)	118 (47.6)
***150–300***	57 (16.7)	26 (23.4)	38 (19.6)	46 (19.2)	45 (18.1)
***> 300***	6 (1.8)	2 (1.8)	3 (1.5)	5 (2.1)	5 (2.0)

* Interpretation of the table (example): Of the 341 total of students who participated, 143 (41.9%) delivered their prevention action in high school. On the 111 students who are willing to change behavior 60 (54.0%) delivered their prevention action in high school

Eighty students (23.5%) reported all the 4 studied positive impacts, 49/341 (14.4%) reported none of them. For students, the probability of reporting satisfaction in their health service experience was associated with the number of perceived positive impacts (*p* < 0.01) (Data shown in supplementary material 3). In the multivariate model, students’ satisfaction was associated with the type of host institution (better in high schools) and the number of beneficiaries reached per week (better when between 50 and 150). There was no significant effect of gender or theme of prevention on students’ satisfaction (Table 3).

**Table 3:** Factors related to students’ satisfaction with their experience of health service

	Unsatisfied students (***n*** = 157, 46%)	Satisfied students (***n*** = 184, 54%)	Univariate analysis	Multivariate analysis
	***N (%)***	***N (%)***	***OR*** ^***raw***^ ***[IC95%]***	***OR*** ^***adjusted***^ ***[IC95%]***
**Gender** (Missing data = 5)				
***Male***	55 (16.1)	50 (14.7)	0.7 [0.4–1.1]*	0.8 [0.5–1.3]
***Female***	102 (30.0)	134 (39.3)	ref	
**E-learning participation**				
***Yes***	94 (27.6)	122 (35.8)	1.3 [0.8–2.0]	
***No***	63 (18.5)	62 (18.2)	ref	
**Place of intervention**				
***Middle School***	8 (2.3)	5 (1.5)	0.3 [0.1–0.9]*	0.6 [0.2–2.4]
***High school***	44 (12.9)	99 (29.0)	ref	Ref
***Universities***	99 (29.0)	60 (17.6)	0.3 [0.2–0.4] *	0.5 [0.3–0.8]
***Social structure***	6 (1.8)	20 (5.9)	1.5 [0.6–3.9]*	1.7 [0.6–5.0]
**Intervention theme**				
***Nutrition***	38 (11.1)	41 (12.0)	Ref	–
***Physical activity***	33 (9.7)	35 (10.3)	1.0 [0.5–1.9]	–
***Addiction***	40 (11.7)	48 (14.1)	1.1 [0.6–2.0]	–
***Sexuality***	46 (13.5)	60 (17.6)	1.2 [0.7–2.2]	–
**Number of beneficiaries per week**				
***< 10***	29 (8.5)	4 (1.2)	0.2 [0.1–0.6]*	0.3 [0.1–0.8]
***10–50***	65 (19.1)	43 (12.6)	ref	ref
***50–150***	41 (12.0)	96 (28.1)	3.5 [2.1–6.0]*	2.7 [1.5–4.8]
***150–300***	18 (5.3)	39 (11.4)	3.3 [1.7–6.4]*	2.0 [0.9–4.4]
***> 300***	4 (1.2)	2 (0.6)	0.8 [0.1–4.3]	0.5 [0.1–3.1]
	***Med (Q1-Q3)***	***Med (Q1-Q3)***	***OR*** ^***raw***^ ***[IC95%]***	***OR*** ^***adjusted***^ ***[IC95%]***
**Mean hours of personal work**	5 (3–7)	5 (3–8)	1.1 [1.0–1.1]*	1.0 [1.0–1.0]

*p value < 0.20, variables with *p* < 0.20 in univariate analysis were included in the multivariate analysis

Concerning the host institutions, 21/23 provided feedback, and of these 19/21 (90%) stated that the prevention action’s content was satisfactory, 10/21 (48%) agreed to welcome students the following year without any modification, 9/21 (43%) agreed with some modifications, and 2/21(9%) did not agree. The modifications they recommended concerned earlier contact with the students, better adaptation of the students to the demands of the host institutions, development of other themes of prevention, strengthening the motivation of the students, improvement of the supports used for delivering prevention action, and improvement of the knowledge of the students. The same reasons were cited by the institutions refusing to repeat the experience.

Regarding the cost, 81 of our students delivered their actions more than 15 km from the Faculty of Medicine and received a compensation of 130 Euros (governed by law), for a total expense of 10,530 Euros. This expenditure, incurred by the universities, is then reimbursed by the social welfare system. In addition, around thirty teachers (from nursing and medical schools) were involved in the delivery of 200 h of face-to-face teaching sessions, dedicated to the public health service of our medical students, in the production of 5 h of e-learning (video) resources and in the coordination of the program, without additional expenses for the university.

## Discussion

Implementation of the public health service program in one Paris faculty shows that inter-sector collaboration (health, education, social) is feasible and enables the implementation of prevention actions targeting various populations. Social inequalities in access to preventive care were tackled by carrying out some prevention actions with vulnerable populations; and the awareness of future health professionals concerning prevention was increased.

Although the experience of students was globally good, training and support were generally considered insufficient. Autonomous project implementation and self-recognition as healthcare professionals are not common experiences for these 3rd year students, so the expectation of preparation and support was important. The application in the 2018/2019 academic year of a decree published in June 2018 posed many organisational challenges. The main ones were: identification of human resources to pilot, teach and supervise the program, planning of the teachers’ and students’ schedule, and recruitment of host institutions. However, among the observed groups, the prevention actions were mostly of good quality, and the peer-to-peer delivery led to soft skills (autonomy, communication, empowerment) development. Being observed may have motivated students to be better organised and generated a Hawthorne (or observer) effect [12]. This raises questions about the balance between the need to strengthen supervision and the necessity to empower our students, taking into account the acceptable workload for them, the faculty teachers or the host-institutions reference persons.

While it has been shown that medical students are inclined - at least as much as other students – towards risky behaviours [13], one third of our students reported a willingness to change their health behaviour after the public health program, and more than two-thirds said they experienced a feeling of ease in addressing prevention issues in future. These results are likely to be improved through a virtuous circle, since a positive association exists between healthy behaviours by medical students and their attitudes towards preventive counselling [14] and the increasing of their knowledge when they carry out peer health education [15].

In this evaluation, students who delivered their actions on a university campus reported less satisfaction than others; this may be explained by the local context of actions. While in schools and social institutions the reference persons were particularly involved in the preparation and the supervision of the action given the vulnerability (age, social situation) of their population, this was less observed in universities. In addition, in universities, the actions were delivered on stands, mainly in entrance halls or forecourts where potential beneficiaries are less easily available and have little time for interaction. Implementation of prevention actions in the universities was therefore more challenging, requiring health students to be more autonomous, and to plan attractive and proactive actions. This may have discouraged less prepared or motivated students.

To our knowledge this kind of national initiative involving a mandatory service involving community-based prevention, and led to a large extent by students acting autonomously, in multiple places, on multiple prevention themes, is singular. The main programs in USA, Switzerland, Brazil, Germany and Wales that have reported positive results in students and beneficiaries in terms of skills and knowledge acquisition rely on voluntary participation; another difference is that the actions previously reported were not built by the students themselves [7, 16–19].

The strength of this study lies in its originality, reporting the results of the first year of implementation of a health promotion service. The methodology used, based on mixed methods and the views of various stakeholders, is particularly adapted to process evaluation of complex interventions [10].

One of the main limitations is related to the beneficiaries’ feedback. Beneficiaries of only 6 student groups’ actions were invited to voluntarily complete the questionnaires, and there is a possible selection bias linked to recruitment in the Social Sciences University where beneficiaries’ participation was based solely on volunteering. However, the collection of the beneficiaries’ opinions of the hundreds of prevention actions was not deemed feasible; and because the questionnaire completion would remain based on volunteering, a high probability of selection bias would subsist. A second limitation relates to the local aspect of the evaluation. A national working group dedicated to the public health service student program, bringing together public health academics, enabled eighteen Faculties of Medicine, including the one studied here, to share their experience. The local organisations are different: the interdisciplinary aspect, the hourly volume of contacts, the teaching method, the specialties of the professionals involved and the theoretical content are all adapted to local constraints and resources. Our local experience therefore does not fully reflect the reality of all the faculties, although a significant number of perceived challenges are shared.

## Conclusion

This study reported the first results of local implementation of the public health service program for students, a French government initiative involving implementation of community-based primary prevention actions led by health sector students. The implementation proved to be feasible and acceptable to all stakeholders. The students and the beneficiaries both reported positive results in terms of knowledge, skills and behaviours. Larger and longer-term studies are needed to confirm the impact of the program on students and beneficiaries.

## Supplementary Information

**Additional file 1 Supplementary material 1.** TIDIeR checklist of health service intervention. Description of the health service program (studied intervention) and details on its local implementation.

**Additional file 2 Supplementary material 2.** Evaluation questionnaires. Questionnaires used for evaluation in the study.

**Additional file 3 Supplementary material 3.** Figure of the percentage of students satisfied of their experience of health service relating to the numbers of positive impacts of health service they reported. Percentage of students satisfied of their experience of health service relating to the numbers of positive impacts of health service they reported (willingness to change behavior; acquisition of knowledge on the theme of the action, acquisition of new skills in prevention, ease of addressing prevention issues in the future).

## Data Availability

The datasets used and/or analysed during the current study are available from the corresponding author on reasonable request.

## Abbreviations

OECD: Organisation for Economic Co-operation and Development
TIDieR: Template for Intervention Description and Replication
