# Supplementary material for: A governmental program to encourage medical students to deliver primary prevention: experiment and evaluation in a French faculty of medicine
Source: BMC Med Educ. 2021 Jan 13;21:47. doi: 10.1186/s12909-020-02472-z (PMC7805043; doi:10.1186/s12909-020-02472-z)
Supplement: Supplementary file 1 — Additional file 1 Supplementary material 1. TIDIeR checklist of health service intervention. Description of the health service program (studied intervention) and details on its local implementation. [file 12909_2020_2472_MOESM1_ESM.pdf]

Supplementary material 1. TIDIeR checklist of health service intervention.

Item	Other † (details)
**1.BRIEF NAME Provide the name or a phrase that describes the intervention.**	The health service is a program set up by Decree No. 2018-472 issued by the French government on June 12, 2018: It is a mandatory service for health students in the French territory; it is included within their initial training and concludes with the realisation of community based prevention action.
**2.WHY Describe any rationale, theory, or goal of the elements essential to the intervention.**	The health service is a major component of the French national health strategy whose objective is to develop health promotion, in line with the international guidance of the World Health Organization. It is based on the following facts:<br>- Non-communicable diseases account for the vast majority of the global burden of disease and are expected to increase further in the context of aging societies<br>- Socially disadvantaged people have a greater risk of exposure to risky behaviours<br>- Evidence suggests that 17% of the total disease burden for all age groups may be associated with risk behaviour in adolescence – which is the period where many behaviours are adopted<br>- Non-communicable diseases can be prevented by effective prevention interventions targeting major risk factors exposition<br>- Health systems do not adequately respond to the current needs of populations, since doctors rarely emphasise prevention as a key tool for changing health outcomes<br>- Medical studies did not adapt until now to the goal of becoming health promoters, with health promotion remaining relatively low priority in medical training programs.
**3. WHAT Materials: Describe any physical or informational materials used in the intervention, including those provided to participants or used in intervention delivery or in training of intervention providers. Provide information on where the materials can be accessed (e.g. online appendix, URL).**	- The health service used teaching material on prevention techniques and questions and on medical knowledge related to risk behaviours and diseases of interest (*material available in French language on demand*).<br>- E-learning sessions: power point presentations were prepared by senior doctors specialised in nutrition, addiction, physical activity or sexual activity. The slides contained theoretical notions about the theme of interest. In addition, students had access to MOOC sessions focused on these same themes.<br>- Workshops on prevention methodology: courses mixing theory and practical exercises delivered by nursing school teachers. The methodological training consists mostly about public health concepts, including practical exercises to teach the students how to create their own prevention tools (questionnaires, presentations…) and how to address specific targets (younger students, migrants and other isolated populations…).<br>- Work sessions of action construction : list of useful resources (websites for accessing prevention tools or goodies)

**4. WHAT Procedures: Describe each of the procedures, activities, and/or processes used in the intervention, including any enabling or support activities.**	- Preliminary phase for the steering committee: Information of all the students by a written information on the health service delivered by email. Recruitment of the places for action (host structures). Elaboration of a specific convention between host structures and faculty, defining the conditions of the stage for medical students. Planning of the students' agenda (course, workshop, times of the actions). Identification of human resources to deliver these courses and supervise students.<br>- Step 1 for students : Training to acquire necessary knowledge and skills. Participation in the interactive “information session” held in an auditorium at the faculty by the steering committee.<br>- Intermediary phase for the steering committee: Attribution of the themes and places of action to the students. Elaboration of groups (of 3 to 4 students).<br>- Step 2 for students : Meeting their group. Development in groups of the preliminary prevention action project, supervised by experts of the prevention theme (work session of action construction). Contact the supervisor of the work session (planned to be an “educational referent”) if any difficulties. Contact and exchange with the referent of the host structure. Adaptation and finalisation of the action project, consensually with the host structure.<br>- Step 3 for students: implementation of the prevention action during one week within the target population in the host structure.<br>- In order to validate the health service, students did not sit any exam, but they have to answer a questionnaire about their opinion on the whole initiative: from the training stage to the actual deployment of their interventions, taking also into account the support received by the faculty.
**5.WHO PROVIDED For each category of intervention provider (e.g. psychologist, nursing assistant), describe their expertise, background and any specific training given.**	The steering committee involved professors teaching in Medicine faculty or school of Nursing, student representatives, and the evaluation team. It was piloted by the head of the educational council of the faculty. The lessons on prevention theory (methodological training sessions) were conducted by nursing school teachers who have experience in prevention training. The medical knowledge was delivered by senior academics in general medicine, addiction, infectiology and nutrition. Junior and senior academics from these same medical disciplines supervised student groups during the development of their actions, as they became their “educational referents”. During the implementation of the action academics stayed available by mail but it was a professional working in the place (host structures) where the prevention action was delivered that served as a referent (in colleges and high schools: science or sports teacher, school nurse, senior education consultant or principal, in universities: professional service provider university medicine or administrative framework responsible for student life, in social structures: responsible health or social professionals). The referents of host structures informed the students

	about the characteristics of the population, the expectations of the structure, the organisation and the form of the intervention to be delivered (places, duration, numbers of beneficiaries). They managed the logistical aspects and in colleges, associations and some high schools they attended the intervention as an observer or mediator.
**6.HOW Describe the modes of delivery (e.g. face-to-face or by some other mechanism, such as internet or telephone) of the intervention and whether it was provided individually or in a group.**	- General information on the health service was sent by mail to all eligible medical students and a face to face information was delivered in auditorium.<br>- For the recruitment of host structures, their directors or representatives were first contacted by e-mail. A phone call or meeting was planned with a member of the steering committee if they agreed to participate or wanted more information.<br>- Theorical knowledge was transmitted online via e-learning.<br>- Workshops on prevention methodology were organised in face-to-face , in small groups of 40 medical students and nursing students.<br>- Work session of action construction was organised in face-to-face, in small groups of around 20 medical students (around 7 groups by classroom).<br>- Medical students were asked to contact the referents in their host structure: by phone or email at least 15 days before the prevention action, and (if requested by host structures) an additional face-to-face meeting was planned before the implementation of the action.<br>- Prevention actions were provided by groups of three to four students. All the interventions were delivered face-to-face, but followed different modalities. The majority of interventions were supervised by local referents in the different host structures : in middle and high schools they were organised as conferences addressed to whole or half class (15 to 30 pupils); whereas in universities medical students delivering the intervention had to catch one or more beneficiaries in circulation areas (entrance hall, cafeteria, classroom's corridors…). In social structures, the beneficiaries were often organised in more reduced groups.
**7.WHERE Describe the type(s) of location(s) where the intervention occurred, including any necessary infrastructure or relevant features.**	Health service was planned in our faculty of medicine.<br><br>The prevention actions prepared by medical students were delivered in middle schools, high schools, universities and social associative structures. These structures could be between 0 and 40 km away of the Faculty of Medicine. Interventions took place in classrooms at the college and high school, in the entrance halls or relaxing places of the universities and in dedicated spaces in the associative structures.
**8.WHEN and HOW MUCH Describe the number of times the intervention was delivered and over what**	The health service took place in the academic year 2018/2019. We used the calendar weeks to explain the chronology of health service (where "week" 1 is the first week of the calendar year, e.g. the week of the 1st of January)

**period of time including the number of sessions, their schedule, and their duration, intensity or dose.**	- Written information on the health service developed by the educational committee of the Faculty and sent to students week 49/53 (year 2018)<br>- Recruitment of places receiving medical students by mail first and then face-to-face or phone. From week 40/53 (year 2018) to week 7/53 (year 2019)<br>- Workshops on prevention methodology (5 methodological lessons, 3 hours each) – on 1 per day on one week. Week 45/53 (year 2018)<br>- Medical knowledge (5 hours of e-learning and 60 hours integrated in the original cursus of students). E-learning available from Week 41/53 (year 2018) non specific lessons (year 2017, year 2018)<br>- Attribution to place and theme for prevention action Week 8/53 (year 2019)<br>- Work sessions of action construction supervised by teachers (1 time, 4 hours) depending of students groups Week 10/53 or Week 11/53 (year 2019)<br>- Contact with the referent of the host structure by mail or phone. depending of students groups between Week 8/53 and 11/53 or between Week 8/53 and Week 14/53 (year 2019)<br>- Implementation of prevention action 5 days full time. depending of students groups Week 13/53 or Week 16/53 (year 2019)<br>- Questionnaires used for our evaluation were send to all the stakeholders on week 20/53 (year 2019)
**9.TAILORING If the intervention was planned to be personalised, titrated or adapted, then describe what, why, when, and how.**	For this first year of implementation, universities were free in their organisation of the health service but should correspond to the decree and related text that stipulated : “The health service is organised within each university in the form of one or more teaching units composed of theoretical and practical training time”; “The health service as defined in Article D. 4071-2 of the Public Health Code, has a total duration of six weeks full-time, with no need for continuity between them, half of which is devoted to realisation of concrete action. This duration includes the duration of the students' theoretical training in prevention, that of the student's personal work, that of the preparation of the prevention action, as well as that of carrying out the action and its evaluation in depending on the specificities of each course and the areas where the action is carried out.”<br><br>Each faculty could then decide how to organise the whole initiative: they could choose the kind of host structures, the topics of public health that would be the theme of actions (they could follow the four themes suggested by French government or add other themes) and how they would train their students for them to be ready to undertake the interventions. The universities could also decide if students had to pass an exam to validate their health service or not.

	In our faculty, all students received the same intervention but do not deliver the same prevention actions (depending of students groups ideas, theme of prevention, kind of host structure and targeted population, request of the host structure).
**10.[‡]MODIFICATIONS If the intervention was modified during the course of the study, describe the changes (what, why, when, and how).**	In our faculty, it was not possible to dedicate half of 6 weeks to the realisation of the action (as written in the decree) given the schedule of our students.<br><br>Recruitment of places to do the prevention actions took longer than imagined; in consequence, the attribution of host structures to groups of students was held later than expected in the beginning.
**11.HOW WELL Planned: If intervention adherence or fidelity was assessed, describe how and by whom, and if any strategies were used to maintain or improve fidelity, describe them.**	NA. This first year experimentation will serve to standardise the intervention for the following year, so we will be able to assess the fidelity later.
**12.[‡]Actual: If intervention adherence or fidelity was assessed, describe the extent to which the intervention was delivered as planned.**	NA
