# Supplementary material for: A governmental program to encourage medical students to deliver primary prevention: experiment and evaluation in a French faculty of medicine
Source: BMC Med Educ. 2021 Jan 13;21:47. doi: 10.1186/s12909-020-02472-z (PMC7805043; doi:10.1186/s12909-020-02472-z)
Supplement: Supplementary file 2 — Additional file 2 Supplementary material 2. Evaluation questionnaires. Questionnaires used for evaluation in the study. [file 12909_2020_2472_MOESM2_ESM.pdf]

Suppl material 2: Evaluation questionnaires

Questionnaire for students

Les questions suivantes portent sur le e-learning
The following questions relate to the e-learning

[Q1] Concernant le E-learning, vous avez regardé :
Tous les cours Tous les cours concernant ma thématique Quelques cours concernant ma thématique (pas tous) Les cours de plusieurs thématiques (mais pas tous) Aucun cours
Regarding E-learning, you have looked at:
All the courses All the courses concerning my theme Some courses concerning my theme (not all) The courses for several themes (but not all) No course

[Q2]Globalement, comment évaluez-vous les E-learning ? :
Très bien Bien Insuffisant Très insuffisant Non applicable, je n'ai pas accédé à aucun E-learning Autre
Overall, how do you assess E-learning?:
Very good Good Insufficient Very insufficient Not applicable, I have not accessed any E-learning Other

[Q3]L'accès aux E-learnings était facile :
D'accord Pas d'accord Non applicable, je n'ai pas accédé à aucun E-learning
Access to E-learning was easy:
Agree Disagree Not applicable, I did not access any E-learning

[Q4]Le contenu de l'enseignement était adapté à votre niveau de connaissances :
D'accord Pas d'accord Non applicable, je n'ai pas accédé à aucun E-learning
The teaching content was adapted to your level of knowledge:
Agree Disagree Not applicable, I have not accessed any E-learning

[Q5]Le contenu de l'enseignement était cohérent et complémentaire avec les autres enseignements de Médecine :
D'accord Pas d'accord Non applicable, je n'ai pas accédé à aucun E-learning
The content of the teaching was consistent and complementary with the other medical lessons :
Agree Disagree Not applicable, I did not access any E-learning

[Q6]Le contenu de l'enseignement était redondant avec d'autres enseignements de vos cours de Médecine :
D'accord Pas d'accord Non applicable, je n'ai pas accédé à aucun E-learning
The content of the teaching was redundant with other lessons in your Medicine courses:
Agree Disagree Not applicable, I did not access any E-learning

[Q7]L'enseignement vous a semblé utile pour la préparation de votre intervention :
D'accord Pas d'accord Non applicable, je n'ai pas accédé à aucun E-learning
The teaching seemed useful to you for the preparation of your intervention:
Agree Disagree Not applicable, I have not accessed any E-learning

[Q8]Le volume horaire attribué à cet enseignement est convenable, la charge de travail associée est acceptable :
D'accord Pas d'accord Non applicable, je n'ai pas accédé à aucun E-learning
The hourly volume allocated to this course is suitable, the associated workload is acceptable :
Agree Disagree Not applicable, I have not accessed any E-learning

[Q9]Le planning et le rythme des cours étaient satisfaisants:
D'accord Pas d'accord Non applicable, je n'ai pas accédé à aucun E-learning
The schedule and pace of the courses were satisfactory:
Agree Disagree Not applicable, I did not access any E-learning

[Q10]Le mode de transmission des connaisances était satisfaisant, la méthode choisie pour délivrer cette formation est idéale :
D'accord Pas d'accord Non applicable, je n'ai pas accédé à aucun E-learning
The mode of transmission of knowledge was satisfactory, the method chosen to deliver this training is ideal:
Agree Disagree Not applicable, I have not accessed any E-learning

[Q11]Il y a eu des problèmes de connexion ou d'autres problèmes techniques pour accéder aux cours :
D'accord Pas d'accord Non applicable, je n'ai pas accédé à aucun E-learning
There were connection problems or other technical problems accessing the courses *:
Agree Disagree Not applicable, I did not access any E-learning

[Q12]Si cet enseignement n'a pas correspondu à vos attentes, merci de nous faire part de vos pistes d'amélioration Veuillez écrire votre réponse ici :
If this teaching did not meet your expectations, please let us know your areas for improvement. Please write your comment here:

Les questions suivantes portent sur les TDs ayant eu lieu en novembre 2018, partagés avec des étudiants infirmiers
The following questions relate to the tutorials that took place in November 2018, shared with nursing students

[A1]A combien de TDs avez-vous assisté ? Seuls des nombres peuvent être entrés dans ce champ. Chaque entrée doit être entre 0 et 5 Veuillez écrire votre réponse ici :
How many tutorials have you attended? Only numbers can be entered in this field. Each entry must be between 0 and 5. Please write your answer here:

[A12]Lequels vous ont semblé les plus pertinents ? *
TD1 : La démarche en santé publique
TD2: Méthodologie de construction d'outils d'enquêtes
TD3: Reccueil et analyse des données
TD4: Construction et élaboration du projet d'action d'éducation en santé
TD5: Méthodes et outils de communication avec le public cible
Which ones did you find most relevant?
1: The public health approach

2: Methodology for constructing survey tools
3: Data collection and analysis
4: Construction and development of the health education action project
5: Methods and tools for communicating with the target audience

[A2]Globalement, comment évaluez-vous les TDs méthodologie dans le cadre du service sanitaire? :
Très adaptés Adaptés Peu adaptés Très peu adaptés
Overall, how do you assess the methodology tutorials within the framework of the health service?:
Very suitable Suitable Little suitable Very little suitable

[A3]Le contenu des TDs était adapté à votre niveau de connaissances:
D'accord Pas d'accord
The content of the tutorials was adapted to your level of knowledge:
Agree Disagree

[A4]Le contenu de l'enseignement était cohérent et complémentaire avec les autres enseignements des études de Médecine:
D'accord Pas d'accord
The content of the teaching was coherent and complementary with the other teaching of medical studies:
Agree Disagree

[A5]Le contenu de l'enseignement était redondant avec d'autres enseignements de vos cours de Médecine :
D'accord Pas d'accord
The content of the teaching was redundant with other lessons in your Medicine cursus lessons:
Agree Disagree

[A6]L'enseignement vous a semblé utile pour la préparation de votre intervention :
D'accord Pas d'accord
The teaching seemed useful to you for the preparation of your intervention:
Agree Disagree

[A7]La quantité de travail demandée dans cet enseignement est acceptable :
D'accord Pas d'accord
The amount of work required in this teaching is acceptable:
Agree Disagree

[A8]Le mode de transmission des connaissances était satisfaisant, la méthode choisie pour délivrer ces informations est idéale :
D'accord Pas d'accord
The mode of transmission of knowledge was satisfactory, the method chosen to deliver this information is ideal :
Agree Disagree

[A9]Le planning et le rythme des cours étaient satisfaisants:

D'accord Pas d'accord
The schedule of the lessons were satisfactory:
Agree Disagree

[A10]Le volume horaire attribué à cet enseignement est convenable :
D'accord Pas d'accord
The time allocated to this course is suitable:
Agree Disagree

[A11]Si ces TDs méthodologie n'ont pas correspondu à vos attentes vous pouvez nous faire part de vos pistes d'amélioration [commentaire]
If these methodological tutorials have not met your expectations, you can tell us about your areas for improvement. [comment]

Les questions suivantes portent sur le TD qui a eu lieu en deux séances: le 5 et le 12 mars.
The following questions relate to the tutorial which took place in two sessions: March 5 and 12.

[C1]Diriez-vous que le TD pré-action a été (plusieurs réponses possibles):
Utile Superflu Rassurant Déstabilisant Suffisamment long Trop long Adapté à vos besoins Autre:
Would you say that the pre-action tutorial was (several answers possible):
Useful Superfluous Reassuring Destabilizing Sufficiently long Too long Adapted to your needs Other:

[C2]Le TD pré-action vous a permis de rencontrer les autres étudiants de votre trinôme :
D'accord Pas d'accord
The pre-action tutorial allowed you to meet other students from your trinomial :
Agree Disagree

[C3]Vous auriez aimé avoir le TD pré-action plus tôt dans l'année :
D'accord Pas d'accord
You would have liked to have had the Pre-Action TD earlier in the year :
Agree Disagree

[C4]Le TD pré-action vous a permis de préparer la totalité de votre action de prévention:
D'accord Pas d'accord
The pre-action tutorial enabled you to prepare all of your preventive action:
Agree Disagree

[C5]Vous auriez aimé avoir plusieurs TDs avec le même format que le TD préaction: toujours avec votre trinôme, toujours avec le même enseignant, axés sur votre thématique, visant votre public cible:
D'accord Pas d'accord
You would have liked to have had several tutorials with the same format as the pre-action tutorial: always with your trinomial, always with the same teacher, focused on your theme, targeting your target audience:
Agree Disagree

[C6]La présence d'un enseignant spécialisé sur votre thématique vous a semblé utile pour la préparation de votre intervention :
D'accord Pas d'accord
The presence of a teacher specializing in your prevention topic seemed useful to you for the preparation of your intervention :
Agree Disagree

[C7]Le fait de pouvoir avoir recours à votre enseignant pour valider l'action que vous avez préparée vous a semblé rassurant :
D'accord Pas d'accord
The fact that you could have recourse to your teacher to validate the action you have prepared seemed reassuring to you:
Agree Disagree

[C8]L'enseignant du TD vous a proposé des liens/outils qui vous ont aidé à construire votre action de prévention :
D'accord Pas d'accord
The TD teacher offered you web links / tools that helped you build your preventive action: Agree Disagree

[C9]Commentaires facultatifs sur le TD pré-action Veuillez écrire votre réponse ici :
Optional Pre-Action TD Comments Please write your response here:

Les questions suivantes portent sur votre semaine d'action de prévention.
The following questions relate to the realisation of your prevention action

[B01]Pendant quelle semaine avez-vous réalisé le stage du service sanitaire ?:
Semaine du 25 mars Semaine du 15 avril
During which week did you complete the health service internship? :
Week of March 25 Week of April 15

[B1]Sur quelle thématique portait l'intervention que vous avez préparée ?:
Alimentation et facteurs de risque cardiovasculaire Activité physique Addictions Santé sexuelle
What theme was the intervention that you prepared?:
Diet and cardiovascular risk factors Physical activity Addictions Sexual health

[B2] Avez-vous sélectionné une sous-thématique que vous avez traitée de façon préférentielle ?: Oui Non
Have you selected a sub-theme that you treated preferentially?: Yes No

[B3]Si oui, laquelle ? Veuillez écrire votre réponse ici :
If yes which one ? Please write your answer here:

[B4]Sur quel type de terrain de stage avez-vous délivré votre intervention ?:
Collège Lycée Etablissement d'enseignement supérieur Structure sociale, association
On what type of internship site did you deliver your intervention? :
College High School Higher education institution Social structure, association

[B5]Quel est le nom de votre terrain de stage ?:
What is the name of your internship site?

[B6] Vis-à-vis des caractéristiques de votre intervention,
1- Quelle était la durée moyenne ? (en minutes)
Regarding the characteristics of your intervention,
What was the average duration? (in minutes)

[B7]2- Quel était l'objectif concret de votre action de prévention ?
Tester les connaissances Renseigner sur les structures d'aide Informer sur les risques Apporter des informations générales sur la thématique Autre:
What was the concrete objective of your preventive action?
Test knowledge Provide information on support structures Provide information on risks Provide general information on the topic Other

[B8]3- Quelle était la tranche d'âge de la population destinataire de votre intervention ?
Veuillez écrire votre(vos) réponse(s) ici : Age minimal Age maximal
What was the age group of the population receiving your intervention?
Please write your answer (s) here: Minimum age Maximum age

[B9]4- Pourriez-vous décrire votre intervention en quelques lignes ? Veuillez écrire votre réponse ici :
Could you describe your intervention in a few lines? Please write your answer here:

[B09]Avez-vous distribué des flyers / goodies / autres documents dans votre intervention ? : Oui Non
Did you distribute flyers / goodies / other documents in your speech? : Yes No

[B010]Si oui, lesquels ? Préservatifs Goodies Flyers Autre:
If yes, which ones ? Condoms Goodies Flyers Other:

[B011]Où les avez-vous obtenus ? Veuillez écrire votre réponse ici :
Where did you get them? Please write your answer here:

[B10] Vis-à-vis de l'encadrement de la part de la faculté (Paris 7) 1- Avez-vous identfiié votre référent comme l'enseignant avec lequel vous avez fait votre TD pré-action ?: Oui Non
Vis-à-vis the supervision from the faculty (Paris 7) 1- Have you identified your referent as the teacher with whom you did your pre-action tutorial? : Yes No

[B11]2- Avez-vous eu recours à ce référent ? : Oui Non
Did you use this referent?: Yes No

[B12]Pour quelle raison ? (Plusieurs réponses sont possibles) * Non applicable (pas de recours à mon référent) Doute sur les modalités pratiques de votre intervention (adaptabilité au public cible, question de communication...) Doute sur le contenu théorique de votre intervention Validation de l'outil choisi pour délivrer votre intervention Autre:
Why ? (Several answers are possible) * Not applicable (no recourse to my referent) Doubt about the practical modalities of your intervention (adaptability to the target audience, communication question ...) Doubt about the theoretical content of your intervention Validation of the tool chosen to deliver your intervention Other:

[B13]3- Diriez-vous que votre référent a été disponible pour répondre à toutes vos demandes ? : Oui Non Non applicable (pas de demandes transmises au référent)

Would you say that your referent was available to answer all your requests?: Yes No Not applicable (no requests sent to the referent)

[B14]4- Croyez-vous que la méthodologie d'accompagnement au service sanitaire mise en place par la faculté (cours, amphi de présentation, encadrement par un référent) vous a permis d'être bien préparépour votre intervention ?: D'accord Pas d'accord
Do you think that the methodology of support to the health service set up by the faculty (courses, presentation lecture hall, supervision by a referent) has enabled you to be well prepared for your intervention? : Agree Disagree

[B15]5- Diriez-vous que vous avez eu le temps nécessaire pour préparer l'intervention ? : D'accord Pas d'accord
Would you say that you had the necessary time to prepare for the procedure? : Agree Disagree

[B16]6- Avez-vous eu recours à des services disponibles à la fac (Paris 7) ?
Did you use the services available at the university

[B17]Commentaires facultatifs sur l'encadrement de la part de la faculté Veuillez écrire votre réponse ici :
Optional faculty comments on coaching Please write your response here:

[B18]Concernant le référent dans votre terrain de stage (CPE, infirmière scolaire, professeur de SVT...) diriez-vous que votre premier échange a eu lieu suffisamment tôt ?:
D'accord Pas d'accord
Regarding the referent in your field of internship (director, teacher ...) would you say that your first exchange took place sufficiently early? :
Agree Disagree

[B20]Diriez-vous que votre référent s'est montré disponible pour vous accompagner dans la préparation de l'intervention (jusqu'à J-1) ?:
D'accord Pas d'accord Non applicable (pas de demandes transmises au référent)
Would you say that your referent was available to assist you in the preparation of the intervention (until D-1)? :
Agree Disagree Not applicable (no requests sent to the referent)

[B19]Merci de préciser combien de semaines (approximativement) avant l'intervention votre groupe a pu avoir un échange (avec une réponse de la part de votre structure d'accueil) avec le référent
Please specify how many weeks (approximately) before the intervention your group could have had an exchange (with a response from your host structure) with the referent.

[B21]Comment évaluez-vous la charge de travail que représente le service sanitaire (hors cours TD et E-learning) ?: Correcte Tolérable Excessive

How do you assess the workload represented by the health service (excluding TD and E-learning courses)? : Correct Tolerable Excessive

[B22]Combien d'heures environ avez-vous consacré à la préparation de votre action de prévention? (Documentation, construction de l'outil, discussion avec les autres intégrants du trinôme...)
Approximately how many hours have you spent preparing for your preventive action? (Documentation, construction of the tool, discussion with the other members of the trinomial ...)

[B23]Commentaires facultatifs sur la préparation de votre action de prévention (Accès aux ressources et aux matériels, démarches administratives...) Veuillez écrire votre réponse ici :
Optional comments on the preparation of your preventive action (Access to resources and materials, administrative procedures, etc.) Please write your answer here:

Les questions suivantes portent sur l'évaluation de votre semaine d'action de prévention.
The following questions relate to the assessment of the realisation of your prevention action

[D1] Vis-à-vis de l'accueil dans le lieu d'intervention, diriez-vous: 1- Que le premier jour de stage vous avez été accuelli par la personne identifiée comme référente dans la structure d'accueil ?: Oui Non
Regarding the arrival in the place of intervention, would you say: 1- That the first day of the internship you were greeted by the person identified as referent in the structure?: Yes No

[D2]2- Que vous avez reçu toutes les instructions nécessaires pour le bon déroulement de votre action de prévention ?:
D'accord Pas d'accord
That you have received all the necessary instructions for the smooth running of your preventive action? :
Agree Disagree

[D3]3- Que le référent de la structure d'accueil a été facilement joignable en cas d'obstacle ? : D'accord Pas d'accord Non applicable (pas d'obstacle rencontré, pas besoin de contacter le référent)
That the referent of the structure was easily reachable in the event of an obstacle?: Agree Disagree Not applicable (no obstacle encountered, no need to contact the referent)

[D4]4- Que votre action s'est bien insérée dans l'offre d'actions déjà existante sur votre terrain de stage ? (Service de Médecine Préventive, infirmière scolaire ou autre): D'accord Pas d'accord Non applicable (il n'y avait pas d'autres actions existantes à ma connaissance)
That your action fit well into the existing action offer on your internship site? (Preventive Medicine Department, school nurse or other): Agree Disagree Not applicable (there were no other actions existing to my knowledge)

[D5]Commentaires facultatifs sur l'accueil dans votre terrain de stage. Veuillez écrire votre réponse ici :
Optional comments on the arrival in your internship site. Please write your answer here:

[D6] Vis-à-vis de l'environnement du lieu d'intervention, diriez vous: 1- Que les emplacements prévus pour le déroulement de votre intervention étaient appropriés ? * : D'accord Pas d'accord
With regard to the environment of the place of intervention, would you say : That the locations planned for the conduct of your intervention were appropriate? : Agree Disagree

[D7]2- Que tous les matériels nécessaires pour la mise en place de votre intervention étaient à votre disposition ?: D'accord Pas d'accord
That all the materials necessary for the implementation of your intervention were at your disposal? *: Agree Disagree

[D8]Commentaires facultatifs sur le lieu d'intervention Veuillez écrire votre réponse ici :
Optional comments on the site of intervention Please write your answer here

[D9] Vis-à-vis de l'action de prévention en elle-même; diriez-vous:
1- Que la méthode que vous avez choisie pour délivrer le message était adaptée ? : D'accord Pas d'accord
With regard to the preventive action itself; would you say: That the method you chose to deliver the message was suitable?: Agree Disagree

[D10]2- A quelles ressources avez-vous eu recours pour répondre aux demandes ou questions des bénéficiaires de vos interventions: Formation spécifique de la fac pour le service sanitaire (E-learning, TD méthodologie...) Support pédagogique (Polycopiés de la fac, collèges de médecine) Encadrement de la part des professionnels rencontrés lors du TD pré-action Recherche sur internet Aucune ressource utilisée Autre:
What resources did you use to answer the requests or questions of the beneficiaries of your interventions: * Specific training of the university for the health service (E-learning, tutorial methodology ...) Educational support (Handouts from the university, colleges of medicine) Supervision by professionals met during the pre-action tutorial Research on the internet No resources used Other:

[D11]3- Combien de personnes ont bénéficié de votre intervention ?: < 10 personnes 10 - 50 personnes 50 - 150 personnes 150-300 personnes > 300 personnes
How many people were exposed to your intervention? : <10 people 10 - 50 people 50 - 150 people 150-300 people> 300 people

[D12]Commentaires facultatifs sur votre action de prévention Veuillez écrire votre réponse ici
Optional comments on your preventive action Please write your response here:

[E01] Selon vous, la prévention doit-elle être intégrée dans le cursus médical ?: Oui Non
In your opinion, should prevention be integrated into the medical curriculum?: Yes No

[E02]Selon vous, la prévention doit-elle être intégrée dans vos cursus dès la troisième année?: Oui Non, c'est trop tôt Non, c'est trop tard
In your opinion, should prevention be integrated into your courses from the third year?: Yes No, it's too early No, it's too late

[E1]D'un point de vue personnel, considérez-vous que votre expérience du service sanitaire tel qu'il a été mis en place cette année a été: Très satisfaisante Satisfaisante Décevante Très décevante
From a personal point of view, do you consider that your experience of the health service as it was set up this year has been: *: Very satisfactory Satisfactory Disappointing Very disappointing

[E2] Pensez-vous que de préparer et délivrer cette intervention vous a permis: 1- D'améliorer vos connaissances sur la thématique de santé que vous avez abordée ?: D'accord Pas d'accord
Do you think that preparing and delivering this intervention has enabled you: 1- Improve your knowledge on the health topic you have discussed? : Agree Disagree

[E3]2- De changer vos comportements de santé ?: D'accord Pas d'accord
To change your health behaviors? : Agree Disagree

[E4]3- D'acquérir des nouvelles compétences en matière de prévention et promotion de la santé ?: D'accord Pas d'accord
To acquire new skills in prevention and health promotion? : Agree Disagree

[E5]5- D'aborder à l'avenir les sujets de prévention plus aisément ?: D'accord Pas d'accord
To tackle prevention topics more easily in the future? : Agree Disagree

[E6]Seriez-vous en faveur de la réalisation du service sanitaire en multidisciplinarité ? (Avec des étudiants infirmiers, kiné, podologues, maïeutique...): Oui Non
Would you be in favor of creating a multidisciplinary health service? (With nursing, physiotherapists, podiatrists, maieutics students...) : Yes No

[E7]Commentaires facultatifs sur le service sanitaire en général:
Optional comments on the health service in general:

Questionnaire for beneficiairies

Avez vous recu plusieurs interventions de prévention dans le cadre du service sanitaire (oui, non)?
Have you received several prevention interventions (yes, no)?

Quel est le thème de prévention de l'intervention que vous évalué ici?
What is the prevention theme of the intervention that you are evaluating here?

Par quel moyen avez vous été informé de cette action de prévention?
How were you informed of this preventive action?

Ces moyens étaient ils suffisants (oui, non)?
Were these information means sufficient (yes, no)?

La localisation de l'action de prévention était elle adapté (oui,non)?
Was the location of the prevention action appropriate (yes, no)?

Concernant le contenu de l'information délivrée dans la séance de prévention diriez vous qu'elle était :
Regarding the content of the information provided in the prevention session, would you say it was

-Adaptée à l'âge (oui, non)
Suitable for your age (yes, no)
-Adaptée au niveau de connaissances (oui, non)
Suitable for the level of your knowledge (yes, no)
-avec des méthodes pédagogiques adaptées (oui, non)
with adapted teaching methods (yes, no)
- d'une durée d'intervention (Suffisante, Trop court, Trop long)
The duration of intervention was (Sufficient, Too short, Too long)
Adaptée pour poser des questions
Suitable for asking questions (yes, no)

L'intervention vous a-t-elle permis :
Did the intervention allow you :

- D'améliorer vos connaissances (oui, non)
To improve your knowledge (yes, no)

- d'être incité à changer de comportement (oui, non)
to be encouraged to change behavior (yes, no)

Comment évaluez-vous l'intervention (Très bien, Bien, Insuffisant, Très Insuffisant)
How would you rate the intervention (Very Good, Good, Insufficient, Very Insufficient)

Commentaire d'appréciation générale
General comment
