# Supplementary material for: A governmental program to encourage medical students to deliver primary prevention: experiment and evaluation in a French faculty of medicine
Source: BMC Med Educ. 2021 Jan 13;21:47. doi: 10.1186/s12909-020-02472-z (PMC7805043; doi:10.1186/s12909-020-02472-z)
Supplement: Supplementary file 3 — Additional file 3 Supplementary material 3. Figure of the percentage of students satisfied of their experience of health service relating to the numbers of positive impacts of health service they reported. Percentage of students satisfied of their experience of health service relating to the numbers of positive impacts of health service they reported (willingness to change behavior; acquisition of knowledge on the theme of the action, acquisition of new skills in prevention, ease of addressing prevention issues in the future). [file 12909_2020_2472_MOESM3_ESM.pdf]

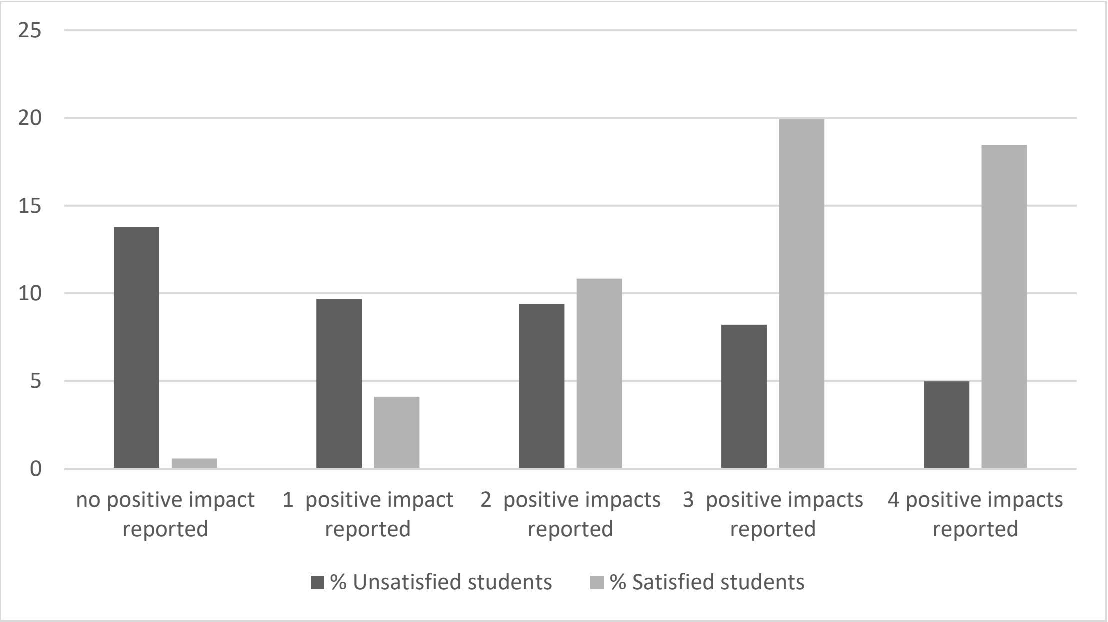

Supplementary material 2. Figure : Percentage of students satisfied of their experience of health service relating to the numbers of positive impacts of health service they reported (willingness to change behavior; acquisition of knowledge on the theme of the action, acquisition of new skills in prevention, ease of addressing prevention issues in the future)
